# The relationship between *Porphyromonas gingivalis* infection and the development of Alzheimer's disease: A scoping review

**DOI:** 10.1177/13872877261456716

**Published:** 2026-06-09

**Authors:** Inês Lopes Cardoso, Maria Inês Guimarães, Elsa Tubiana, Sandra Gavinha

**Affiliations:** 1 RISE-Health, Faculty of Health Sciences, Faculty of Health Sciences, 386392 Fernando Pessoa University, Fernando Pessoa Teaching and Culture Foundation, Porto, Portugal; 2 Faculty of Health Sciences, 386392 Fernando Pessoa University, Fernando Pessoa Teaching and Culture Foundation, Porto, Portugal; 3 FP-I3ID, Institute of Investigation, Innovation and Development, FP-BHS, Biomedical and Health Sciences, University Fernando Pessoa, Porto, Portugal; 4 Escola de Medicina e Ciências Biomédicas (EMCB), Fernando Pessoa Teaching and Culture Foundation, Gondomar, Portugal; 5 INMLCF, I. P., North Delegation - National Institute of Legal Medicine and Forensic Sciences, I. P., Porto, Portugal; 6 CEISUC, Centre of Investigation in Technologies and Centre for Health Studies and Research of the University of Coimbra, Coimbra, Portugal; 7 CIBB- Centre for Inovation in Biomedicine and Biotechnology, Coimbra, Portugal

**Keywords:** Alzheimer's disease, neurodegeneration, periodontal disease, *Porphyromonas gingivalis*

## Abstract

**Background:**

Alzheimer's disease is the leading cause of dementia and constitutes a major public health problem. Recent research suggests that certain chronic infections, particularly periodontal infections, may play a role in the development or progression of this disease. Among the bacteria involved in periodontal disease, *Porphyromonas gingivalis* has attracted particular attention from researchers.

**Objective:**

In this way, the aim of this thesis, conducted in the form of a scoping review, was to analyze existing scientific data on the relationship between *Porphyromonas gingivalis* infection and Alzheimer's disease.

**Methods:**

To achieve this, a literature search was conducted in several scientific databases leading to the selection of fourteen studies that met the inclusion criteria.

**Results:**

Some of the selected studies have shown the presence of *Porphyromonas gingivalis* or its virulence factors in the brain tissues of patients with Alzheimer's disease. Experimental studies also indicate that this bacterium can promote certain mechanisms involved in neurodegeneration, namely inflammation and accumulation of amyloid-β.

**Conclusions:**

Selected studies point to the existence of an association between exposure to periodontal bacteria and an increased risk of developing Alzheimer's disease.

## Introduction

Alzheimer's disease (AD) is the leading cause of dementia, manifesting as progressive impairments in memory, language and executive function, associated with amyloid deposits, tau protein abnormalities and chronic inflammation of the brain.^1^

In addition to known genetic factors, a growing body of research suggests that modifiable factors, including chronic infections and systemic inflammation, may play an important role in the development and progression of AD.^2^

In this context, oral health, and periodontitis in particular, has attracted considerable attention. Periodontitis is a chronic infection of the gums and periodontal tissues, common in adulthood, which repeatedly exposes the body to bacteria and their toxins.^3^ Among these bacteria, *Porphyromonas gingivalis* (*P. gingivalis* or Pg) is considered a key pathogen as it is capable of disrupting the local immune response, destroying periodontal tissues and spreading beyond the oral cavity.^3^ Epidemiological studies have shown that the presence of antibodies against periodontal bacteria, including *P. gingivalis*, is associated with a higher risk of developing dementia, including AD, particularly in individuals who also have other chronic infections such as hepatitis C and herpes simplex virus type 2 (HSV-2).^2^

More recent studies have focused on what occurs in the brain itself. *P. gingivalis* and its toxic proteins, gingipains, have been detected in the brains of patients with AD, and an association has been identified between their presence and the severity of amyloid lesions and tau protein.^1^ *In vitro* experiments show that the bacterium *P. gingivalis* can infect human neurons derived from induced pluripotent stem cells (iPSCs), persist within these cells and induce changes similar to those observed in AD, such as tau protein degradation, increased phosphorylation of tau and the loss of synapses.^4^ In experiments using rats as models for the induction of experimental periodontitis or oral infection with *P. gingivalis*, researchers suggest a link between periodontal inflammation, increased amyloid deposits in the brain, changes in microglia and worsening of markers of neurodegeneration.^1,3^

In light of the above, this thesis, in the form of a systematic review, forms part of this rapidly expanding field of research. The aim is to provide a structured overview of the relationship between *P. gingivalis* and AD, focusing on clinical and epidemiological data, *postmortem* study results, cellular and animal models, as well as the first therapeutic leads targeting gingipains.^1–4^ By searching and organizing the data available in the literature, this study aims to clarify the extent to which *P. gingivalis* might contribute to the development or progression of AD, addressing the main limitations of current studies and how this evidence can inform preventive strategies focused on periodontitis control and oral health.^1,2^

## Methods

A research protocol was developed based on the Joanna Briggs Institute (JBI) model,^5–7^ which led to the formulation of the following research question: What is the relationship between *P. gingivalis* infection and the development of AD? Thus, the acronym PCC used was that presented in Table 1.

**Table 1.** PCC strategy (population, concept, context).

Population	Individuals with *porphyromonas gingivalis* infection
Concept	The relationship between infection/inflammation caused by *Porphyromonas gingivalis* and the development of Alzheimer's disease
Context	Biomedical research and clinical studies on the oral microbiome, inflammation and neurodegeneration

For the final review, the items identified in the reports prepared to guide systematic reviews and meta-analysis extensions (PRISMA-ScR) were used. This protocol was registered on the OSF platform (https://osf.io/z6wc4/overview (accessed on 5 January 2026)).

### Inclusion and exclusion criteria

#### Inclusion criteria

Original studies published in peer-reviewed scientific journals that examine the relationship between *P. gingivalis* and AD; articles written in English, Portuguese, French or Spanish; studies involving human or animal models.

#### Exclusion criteria

Articles for which the full text is not available; publications in languages other than those specified in the inclusion criteria; studies not related to *P. gingivalis* or AD; editorials, letters, conference abstracts or commentaries without primary data; review articles; studies focusing exclusively on other bacteria or other neurodegenerative diseases, surveys, and studies on systemic diseases or treatments.

### Search strategy

The search strategy was designed by two reviewers and reviewed by a third specialist reviewer, using the Peer Review of Electronic Search Strategies (PRESS) checklist.^8^

For this scoping review, a search was conducted in the following databases: PubMed, ScienceDirect, Web of Science, CINAHL Plus with Full text and MEDLINE with Full text. The search strategy recommended by the JBI was implemented.

A preliminary search of the databases was carried out to identify the keywords and index terms used in publications relating to the topic. This enabled the development of the search strategy for each database, as presented in Table 2. This search was conducted on 23 March 2025.

**Table 2.** Search strategy for each database.

Database	Articulation of keywords	Number of articles
PubMed	*(“Porphyromonas gingivalis” OR “P. gingivalis”) AND (“Alzheimer's disease” OR “Alzheimer disease” OR “AD”) AND (infection OR inflammation OR “oral microbiome” OR “periodontal disease”)*	204
ScienceDirect	*(“Porphyromonas gingivalis” OR “P. gingivalis”) AND (“Alzheimer's disease” OR “Alzheimer disease” OR “AD”) AND (infection OR inflammation OR “oral microbiome” OR “periodontal disease”)*	1759
Web of Science	*((((((((ALL=(Porphyromonas gingivalis)))) AND ALL=(Alzheimer's disease))) AND ALL=(infection))))*	150
CINAHL Plus with Full text	*(“Porphyromonas gingivalis” OR “P. gingivalis”) AND (“Alzheimer's disease” OR “Alzheimer disease” OR “AD”) AND (infection OR inflammation OR “oral microbiome” OR “periodontal disease"*	33
MEDLINE with Full text	*(“Porphyromonas gingivalis” OR “P. gingivalis”) AND (“Alzheimer's disease” OR “Alzheimer disease” OR “AD”) AND (infection OR inflammation OR “oral microbiome” OR “periodontal disease”)*	272

A review of the reference lists of all included articles was carried out to assess the possibility of including additional articles. The results of the electronic search were exported to Rayyan^®^.^9^ The software was developed by Rayyan Systems Inc. of Cambridge, MA, USA. The AI-driven software was not used to select the articles; it was used solely as a support tool to gather all the articles found in the various databases used, identify duplicates and select the articles to be included. The identified duplicates were removed.

### Selection, analysis, and studies presentation

After removing duplicates, two reviewers independently screened the titles and abstracts against the inclusion and exclusion criteria to decide whether to include the articles in the scoping review. During this first screening stage, titles and abstracts were assessed to determine their relevance to both *P. gingivalis* and AD. Studies were considered potentially eligible if the title or abstract explicitly mentioned: a) *P. gingivalis* or clearly referred to this pathogen as a periodontal keystone bacterium; b) AD, cognitive impairment, dementia, or key pathological features of this disorder. Abstracts were judged as relevant if they suggested a direct or indirect association between *P. gingivalis* infection and AD-related outcomes.

The full-text articles from studies with potential for inclusion in this review were assessed independently by the same two reviewers. Any uncertainties or disagreements encountered were discussed with a third reviewer, in accordance with the Peer Review of Electronic Search Strategies (PRESS) checklist.^8^

**Figure 1.**
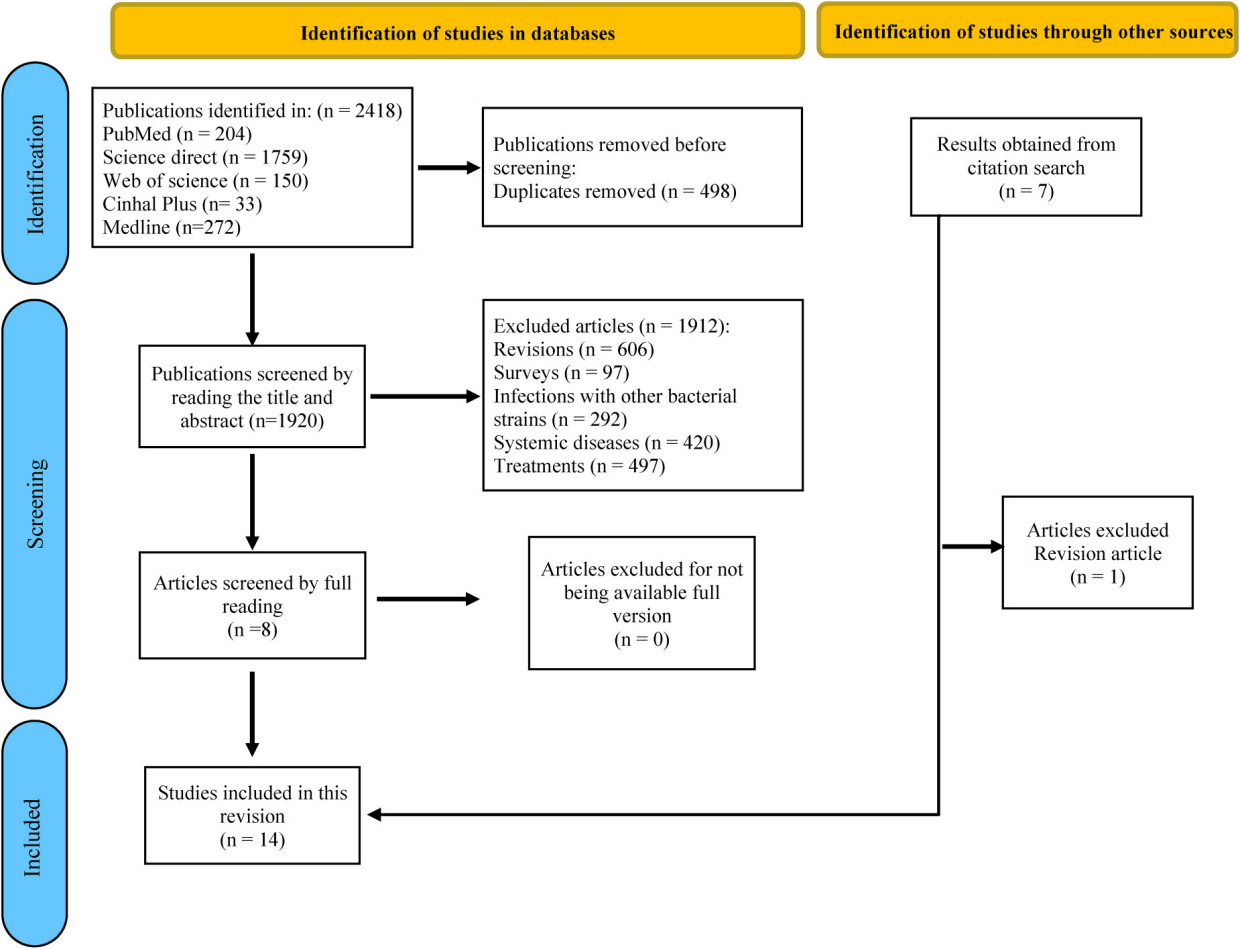
Fluxogram of the paper selection process, adapted from the PRISMA 2000 flow diagram.^10^

The assessment and selection of articles was carried out in two stages: firstly by reading the title and abstract, and secondly by reading the full text. This methodological strategy is illustrated in the PRISMA flowchart (Figure 1).

After reading the full-text articles included in this scoping review, data were extracted in accordance with the objectives and research questions of this review, using the tool proposed by the Joanna Briggs Institute,^5–7^ covering the following relevant information: title, author(s), year of publication, country of origin, study type, objective(s) and main results.

In the first phase of the search, filters were applied, resulting in 2418 articles, of which 498 duplicates were identified and removed. Of the remaining 1920 articles, the initial screening by title and abstract excluded 1912 for the reasons presented in Figure 1. Eight articles were selected for full-text review. A manual search of the reference lists identified 7 additional articles, of which 1 was rejected. Ultimately, 14 articles were included in this scoping review (Figure 1).

## Results

Most of the selected publications are *in vitro* studies, with one *in vivo* study and three comparative studies. Table 3 provides details of the articles included in this review, as well as results of those studies.

**Table 3.** Description of observed results of selected articles.

Author (year)	Title	Type of study	Study objective	Methodology	Results
Kamer et al.^12^	TNF-α and antibodies to periodontal bacteria discriminate between Alzheimer's disease patients and normal subjects	*In vitro*	To assess whether plasma TNF-α and IgG antibodies against periodontal bacteria (Pg, Tf and Aa) can distinguish patients with AD from cognitively normal elderly individuals.	– Sample: 18 patients with AD; 16 cognitively normal individuals. – Analyses: determination of *APOE* genotype in blood samples; measurement of plasma IgG against Pg, Tf and Aa by ELISA in fasting plasma samples; measurement of plasma cytokines (TNF-α, IL-1β and IL-6) by multiplex assay; statistical analysis using SPSS.	– 72% of patients with AD are positive for ≥1 periodontal IgG; 38% of controls are positive for ≥1 periodontal IgG. – TNF-α levels are significantly higher in patients with AD compared with controls. – A positive result for TNF-α or IgG against Pg/Tf/Aa allows patients with AD to be distinguished from healthy individuals. – The combination of TNF-α and a positive IgG result provides greater specificity (≈ 81.3%) and sensitivity (≈ 83.3%).
Stein et al.^13^	Serum antibodies to periodontal pathogens are a risk factor for Alzheimer's disease	*In vitro*	To examine serum IgG levels against periodontal disease-associated bacteria in participants who went on to develop AD, compared with individuals in the control group.	– Sample: 81 cognitively normal individuals who developed mild cognitive impairment or AD during follow-up; 77 cognitively normal subjects who remained so during follow-up. – Analyses: measurement of serum IgG against Pg, Tf, Aa, Td, Cr, Fn and Pi; statistical analysis using linear regression.	– Significantly elevated levels of IgG against Fn and Pi in patients with AD compared with controls, prior to the diagnosis of neurological changes. – Significantly elevated levels of IgG against Td and Pg at baseline. – Samples obtained years prior to the clinical diagnosis of AD or mild cognitive impairment, whilst the individuals were still cognitively normal—the observed increases cannot be attributed to secondary effects of the AD process, such as malnutrition or other neglect associated with dementia.
Poole et al.^18^	Determining the presence of periodontopathic virulence factors in short-term postmortem Alzheimer's disease brain tissue	*In vitro*	To identify the main bacteria responsible for periodontal disease (Td, Tf and Pg) and/or bacterial components present in the brain tissue of patients with AD 12 h after death.	– Sample: *postmortem* brain tissue from 10 patients with AD and 10 controls. – Analyses: immunofluorescence using antibodies against Td, Tf and Pg on brain tissue sections; identification of LPS and gingipains in protein extracts by Western blot; statistical analysis using the Mann-Whitney U test.	– Four out of ten brains from patients with AD showed LPS staining by immunofluorescence. – Pg LPS was identified in all four immunofluorescence-positive samples. – Controls were consistently negative. – LPS from periodontal bacteria may reach the brains of patients with AD during their lifetime.
Noble et al.^15^	Serum IgG antibody levels to periodontal microbiota are associated with incident Alzheimer disease	*In vitro*	To investigate whether serum antibodies (IgG) against periodontal bacteria, including Pg, can predict the incidence of AD.	– Sample: 110 patients with AD; 109 cognitively normal subjects. – Analyses: measurement of serum IgG against Pg, Tf, Aa, Td, Cr, En and An; statistical analysis using t-tests and chi-squared tests.	– Elevated levels of IgG against Cr (69%), against Tf (63%), against Td (53%), against Pg (23%), against En (19%), against Aa (11%) and against An (10%). The proportions did not differ between patients and controls. – High levels of IgG against An—increased risk of AD. – High levels of IgG against En—reduced risk of AD.
Carter et al.^19^	The *Porphyromonas gingivalis*/host interactome shows enrichment in GWASdb genes related to Alzheimer's disease, Diabetes and Cardiovascular diseases	Comparative study	To analyze the relationship between the Pg/host interactome and the genes identified in genome-wide association studies (GWAS) for cardiovascular disease (CVD), type 2 diabetes mellitus (T2DM), AD and other chronic diseases.	– Sample: data from GWASdb and, in some cases, from the NCBI/EBI GWAS database. – Analyses: gene expression data from periodontitis or Pg microarrays compared with microarray datasets from the hippocampus in AD and/or from carotid artery plaques.	– Host genes from the Pg interactome were significantly enriched in genes listed in the GWASdb that are associated with cognitive disorders, AD and dementia, and the comorbid conditions T2DM, obesity and CVD. – The Pg/host interactome was enriched in GWAS genes from the most rigorous NCBI-EBI database for AD, atherosclerosis and T2DM. – Genes dysregulated in periodontal tissue or in Pg-infected macrophages corresponded to those in the hippocampus with AD or atherosclerotic plaques. – Data suggest important gene-environment interactions between Pg and susceptibility genes or alterations in gene expression in conditions where periodontal disease is a contributing factor.
Laugisch et al.^17^	Periodontal pathogens and associated intrathecal antibodies in early stages of Alzheimer's disease	*In vitro*	To investigate the presence of periodontal pathogens in the early stages of dementia and the intrathecal production of specific antibodies against these bacteria in patients with AD compared with those with other forms of dementia (DEM-noAD).	– Sample: 40 patients—20 with AD and 20 with other forms of dementia. – Analyses: recording of periodontal indices; quantification of Aβ1-42 and T-tau in CSF; quantification of IgG antibodies against Pg, Aa and Treponema spp. in serum and CSF; detection and quantification of Pg, Tf and Td by qPCR; quantification in serum and CSF of antibodies against Pg, Td and Aa, and of albumin; determination of the antibody/albumin ratio; statistical analysis using SPSS.	– Significantly lower levels of Aβ1-42 in CSF in patients with AD than in patients with non-AD MSD. – No difference between the groups in the presence of periodontal destruction and inflammation. – Pg, Tf and Treponema spp. were present in over 50% of subgingival biofilm samples, but not in serum or CSF. – Elevated levels of anti-pathogen antibodies in the CSF of 7 patients with AD and 9 with MSD-non-AD—compared with serum, this highlights the possibility of an intrathecal immune response to pathogens. – No significant difference in antibody levels against selected bacteria in CSF and serum between the groups. – Association between T-tau levels in the DA group and serum levels of anti-Pg antibodies.
Bennett et al.^20^	RNA sequencing reveals small and variable contributions of infectious agents to transcriptomes of postmortem nervous tissues from Amyotrophic Lateral Sclerosis, Alzheimer's disease and Parkinson's disease subjects, and increased expression of genes from disease-activated microglia	*In vitro*	To assess the contribution of infectious agents to the transcriptome of *postmortem* nervous tissue from patients with AD, Parkinson's disease (PD) and amyotrophic lateral sclerosis (ALS).	– Samples: cervical spinal cord from individuals with ALS; frontal cortical grey matter from individuals with AD; ventral midbrain from individuals with PD; controls. – Analyses: sequencing of RNA extracted from the samples; alignment of the sequences with transcriptomes from Pg, Td, and others.	– < 0.1% of transcriptomes aligned with the genomes of B. microti, B. burgdorferi, Td and Pg. – Higher percentages aligned with the genomes of T. gondii and Trichinella spp. – In AD samples, but in no others, the percentages of primary aligned transcriptomes approached significance as they were higher in AD compared with controls for B. burgdorferi and Pg. – Detection of significant changes in the expression of disease-associated microglial genes in post-mortem tissues from AD and ALS, but not from PD.
Dominy et al.^1^	Porphyromonas gingivalis in Alzheimer's disease brains: evidence for disease causation and treatment with small-molecule inhibitors	*In vitro*	To assess whether Pg infection contributes to the pathogenesis of AD through the secretion of gingipains, thereby promoting neuronal damage.	– Samples: *postmortem* brain tissue from 10 patients with AD; brain tissue from control subjects (without AD); gingival tissue, CSF and saliva from patients with AD and controls. – Analyses: identification of gingipains, tau protein and ubiquitin in tissues by immunohistochemistry; analysis of Pg and H. pylori by qPCR in brain tissue, CSF and saliva; sequencing of amplified Pg DNA; quantification of Aβ1-42 and TNF-α by ELISA in brain tissue; infection of mice with Pg.	– Presence of Pg in the brains of patients with AD. – Presence of gingipains in the brains of patients with AD, with levels correlating with tau and ubiquitin pathology. – Colonization of the brain and increased production of Aβ1-42 in mice orally infected with Pg.
Beydoun et al.^16^	Clinical and bacterial markers of periodontitis and their association with incident all-cause and Alzheimer's disease dementia in a large national survey	Comparative study	To assess the association between periodontal and bacterial clinical parameters and the incidence of dementia (all causes and AD) and mortality from AD in middle-aged and older adults in the US.	– Sample: data from the third National Health and Nutrition Examination Survey (NHANES III, 1988−1994) linked longitudinally to the National Mortality Index and Medicare data up to 1 January 2014, with a follow-up period of up to 26 years. – Analyses: assessment of clinical periodontal markers (attachment loss, periodontal pocket depth); determination of IgG for various periodontal pathogens; use of Cox proportional hazards models adjusted for multiple variables specific to sex and age.	– ≥65 years—incidence and mortality from DA associated with greater probing depth and IgG titres against Pg, Cr and *Prevotella* spp. – Incidence of DA associated with the combination of C. rectus and Pg. – Risk of mortality from DA associated with a high IgG titer against Pg, *Prevotella intermedia*, *Prevotella nigrescens*, *Fusobacterium nucleatum*, *C. rectus*, *Streptococcus intermedius*, *Capnocylophaga Ochracea* and *P. melaninogenica*.
Haditsch et al.^4^	Alzheimer's disease-like neurodegeneration in *Porphyromonas gingivalis* infected neurons with persistent expression of active gingipains	*In vitro*	To demonstrate that Pg is capable of invading and persistently infecting mature human neurons in vitro, with continuous expression of active gingipains, and to assess whether this infection induces neurodegenerative changes similar to those seen in AD.	– Sample: neurons derived from human induced pluripotent stem cells (iPSCs). – Treatment: infection of the neurons with Pg for 24, 48 and 72 h. – Analyses: characterization of the infection by electron and confocal microscopy; quantification of colony-forming units; assessment of gingipain expression by RT-qPCR; determination of gingipain activity by colorimetric enzyme assay; analysis of total Tau, synapsin and gingipains by Western blot; determination of phospho-Tau by ELISA; statistical analysis using GraphPad Prism.	– Identification of neurons infected with Pg, with the pathogen remaining free in the cytoplasm or within lysosomes. – Neurons initially survived but exhibited cell death that was dependent on the duration of infection. – Accumulation of autophagic vacuoles and multivesicular bodies, marked degradation of tau with increased phosphorylation, reduced density of presynaptic buttons and signs of synaptic loss, as a result of the infection. – “AD-like” neurodegenerative phenotype.
Kantarci et al.^3^	Microglial response to experimental periodontitis in a murine model of Alzheimer's disease	*In vivo*	To investigate how experimental ligature-induced periodontitis modulates the microglial response and Aβ pathology in an animal model of AD.	– Sample: 5xFAD and wild-type mice. – Treatment: induction of periodontitis by ligating molars (ligature-induced periodontitis). – Analyses: assessment of alveolar bone loss and osteoclasts (TRAP); quantification of soluble/insoluble Aβ in the brain; immunohistochemistry for Aβ42 and microglia (Iba1); analysis of brain cytokines.	– 5xFAD mice exhibited greater baseline bone loss than wild-type mice; ligation caused additional bone loss in both groups. – Periodontitis increased insoluble Aβ42 in 5xFAD mice and altered the microglial response.
Fu et al.^14^	Oral microbiome and serological analyses on association of Alzheimer's disease and periodontitis	*In vitro*	To investigate the association between AD and periodontitis by assessing periodontal status, serum markers (including anti-Pg) and the composition of the oral microbiome.	– Sample: 20 patients with AD and 20 healthy controls. – Analyses: assessment of clinical periodontal markers (attachment loss, periodontal pocket depth, plaque and gingival indices, number of teeth); serum levels of Aβ42, Tau, pTau, inflammatory cytokines, hsCRP, triglycerides and anti-LPS antibodies from Pg; analysis of the salivary microbiome by sequencing; statistical analysis using t-tests, chi-square tests and binary logistic regression with SPSS.	– Greater loss of dentin and significantly higher serum levels of Tau, hsCRP and anti-Pg LPS antibodies in patients with AD compared with controls. – No statistically significant increase in pTau and Aβ42. – pTau showed a positive correlation with the titre of anti-Pg LPS antibodies. – Higher abundance of certain oral bacterial species (*Capnocytophaga* sp., *Eubacterium infirmum*, *Prevotella buccae*, *Selenomonas artemidis*) in AD patients.
Zhang et al.^11^	Porphyromonas gingivalis msRNA P.G_45033 induces amyloid-β production by enhancing glycolysis and histone lactylation in macrophages	*In vitro*	To elucidate how Pg microRNA (Pg_45033) induces the production of Aβ in macrophages, by investigating the role of glycolysis and histone lactylation, in order to better understand the link between periodontitis and AD.	– Sample: human U937 monocytes. – Treatment: induction of monocyte differentiation into macrophages and transfection of the macrophages with P.G_45033 mRNA (compared with other Pg mRNAs and a control). – Analyses: measurement of glucose consumption and pyruvate and lactate production; determination of glycolytic enzyme activity; analysis of glucose metabolism gene expression by PCR array; measurement of acetylated histone levels by Western blot; quantification of intracellular Aβ by immunofluorescence and in culture medium by ELISA, with and without glycolytic inhibition by 2-DG; statistical analysis using SPSS.	– Significant increase in glycolysis in macrophages expressing P.G_45033 mRNA: ↑ glucose consumption; ↑ lactate/pyruvate; ↑ activity of hexokinase, pyruvate kinase and lactate dehydrogenase; ↓ pyruvate dehydrogenase; increased expression of glycolytic genes. – Increased histone acetylation following transfection with P.G_45033. – Significant increase in Aβ production (intracellular and in culture medium). – 2-DG attenuated the effects, indicating that P.G_45033 induces Aβ via enhanced glycolysis and histone acetylation in macrophages.
Beydoun et al.^2^	Infection burden, periodontal pathogens, and their interactive association with incident all-cause and Alzheimer's disease dementia in a large national survey	Comparative study	To examine how the overall burden of infections and periodontal pathogens or markers are associated with the risk of all-cause dementia and Alzheimer's disease in a US national sample.	– Sample: 2997 participants in the Third National Health and Nutrition Survey linked to CMS-Medicare (aged ≥ 45 years; ≤ 30 years of follow-up). – Analyses: serology for multiple infectious agents (viruses, bacteria, parasites) to calculate the overall burden of infections; IgG against 19 periodontal pathogens; clinical periodontal data (probing depth, attachment loss); risk models for global dementia and Alzheimer's disease.	– Hepatitis C and herpes simplex virus type 2 are strongly associated with an increased risk of global dementia. – Positive IgG antibodies to Pg and Streptococcus oralis are associated with an increased risk of AD at high levels of overall infection burden. – Certain combinations of periodontal pathogens, in conjunction with a high overall infection burden, increased the risk of dementia, particularly among ethnic minorities. – Pocket depth was associated with dementia, particularly at low levels of overall infection burden.

The presence of *P. gingivalis* and its virulence enzymes (gingipains) has been observed in the brains of patients with AD.^1^ This study demonstrated that infecting mice with this bacterium leads to increased amyloid-β (Aβ) production. Furthermore, the use of gingipain inhibitors reduces inflammation and neurodegeneration in these patients. In line with these results, Haditsch et al.^4^ demonstrated the ability of *P. gingivalis* to infect neurons in vitro, leading to the death of these cells. The infected neurons exhibited a neurodegenerative phenotype similar to that seen in AD.

In another *in vitro* study, *P. gingivalis* led to significant alterations in various enzymes involved in glucose metabolism in transfected macrophages, resulting in increased Aβ production.^11^

These findings are supported by the study by Kantarci et al.^3^ who, using an animal model of AD, demonstrated that experimental induction of periodontitis leads to an increase in insoluble Aβ and alters microglial activation, suggesting a link between periodontal inflammation and neuroinflammation in AD.

Kamer et al.^12^ observed that patients with AD had higher levels of TNF-α and IgG antibodies against certain periodontal bacteria. The combination of these markers made it possible to distinguish patients with AD from cognitively healthy individuals. Indeed, elevated levels of antibodies against various periodontal pathogens were observed in some individuals several years prior to the diagnosis of AD.^13,14^ Similarly, Noble et al.^15^ and Beydoun et al.^16^ demonstrated that elevated serum levels of IgG against certain periodontal bacteria are associated with a higher risk of developing AD. Antibodies against periodontal bacteria were detected by Laugisch et al.^17^ in the cerebrospinal fluid of patients with AD, suggesting an intrathecal immune response associated with periodontal infections.

In addition to antibodies, lipopolysaccharides (LPS) from *P. gingivalis* were detected in the postmortem brain tissue of patients with AD.^18^ These LPS were absent in individuals in the control group, suggesting that bacterial virulence factors may reach the brain and contribute to the inflammatory processes associated with AD. Patients with AD also had higher levels of Tau and high-sensitivity C-reactive protein (hs-CRP) in serum.^14^ This study observed a positive correlation between the presence of anti-*P. gingivalis* antibodies and the biomarker phosphorylated Tau protein (pTau), suggesting a link between periodontal infection and the mechanisms underlying the development of AD.

Some studies suggest that infection with *P. gingivalis* may influence genetic mechanisms involved in the pathophysiology of AD^19^ and may contribute to the expression of genes related to microglial activation in the *postmortem* brain tissues of patients with neurodegenerative diseases.^20^

Beydoun et al.^2^ demonstrated that IgG antibodies against *P. gingivalis* and *Streptococcus oralis* bacteria are associated with an increased risk of AD in individuals with a high bacterial load.

## Discussion

The studies analyzed in this scoping review employ different methodological approaches, including epidemiological studies, analyses of *postmortem* brain tissue, and in vitro and in vivo experimental models.

Overall, these studies show that chronic exposure to periodontal bacteria may contribute to certain mechanisms involved in neurodegeneration. Indeed, several of the epidemiological studies analyzed demonstrated that individuals with high levels of antibodies against periodontal bacteria had a higher risk of developing AD. This was observed by Kamer et al.^12^ in patients with this disease, who had higher concentrations of TNF-α and antibodies against certain periodontal bacteria than cognitively healthy individuals. These results suggest that systemic inflammation associated with periodontal disease may play a role in the pathological processes of AD.

Other studies also suggest that exposure to periodontal bacteria may precede the onset of neurological symptoms. Stein et al.^13^ demonstrated that high levels of antibodies against various periodontal pathogens were already present in individuals several years prior to the clinical diagnosis of AD. This suggests that periodontal infection may represent a potential risk factor, rather than a consequence of the disease.

Similar findings were also reported by Noble et al.,^15^ who observed that elevated levels of IgG antibodies against periodontal bacteria were associated with an increased risk of developing AD over time.

Some studies have also attempted to directly identify the presence of periodontal bacteria or their components in the brain. Poole et al.^18^ detected bacterial lipopolysaccharides in *postmortem* brain tissue from patients with AD. Similarly, Dominy et al.^1^ identified the presence of *P. gingivalis* and its virulence proteins, gingipains, in the brains of patients with AD. These authors also observed that these proteins were associated with alterations in tau protein, which could indicate a role for the bacterium in neurodegenerative mechanisms.^1^

These results suggest that *P. gingivalis* may contribute to AD by interacting with certain susceptibility genes identified in genetic studies. As suggested by Carter et al.,^19^ periodontal infection may influence the expression of various genes involved in immune and inflammatory responses, which may promote the neurodegenerative processes observed in AD.

Similar findings were reported by Beydoun et al.,^16^ who investigated the association between periodontal pathogens and the risk of dementia in a large cohort from the NHANES study. The authors demonstrated that higher levels of IgG antibodies directed against *P. gingivalis* were associated with an increased risk of AD, as well as mortality related to this condition in older adults. These observations reinforce the hypothesis that periodontal infections may contribute to the processes involved in neurodegeneration.

Findings were reported by Fu et al.,^14^ who investigated the association between AD, the oral microbiome and certain serum biomarkers. The authors demonstrated that patients with AD had higher serum levels of Tau, hs-CRP and antibodies directed against *P. gingivalis* LPS. Furthermore, certain bacterial species, such as *Eubacterium infirmum* and *Prevotella buccae*, were more abundant in patients with AD, suggesting that alterations in the oral microbiome and systemic inflammation could contribute to the pathophysiological mechanisms of the disease.

Beydoun et al.^2^ investigated the association between overall infectious burden and periodontal pathogens in the NHANES III cohort. The authors demonstrate that certain infections, notably hepatitis C and HSV-2, are associated with an increased risk of dementia. Furthermore, high levels of antibodies against *P. gingivalis* and *Streptococcus oralis* are associated with an increased risk of AD in individuals with a high infectious burden. These results suggest that the interaction between systemic infections and periodontal pathogens may contribute to the mechanisms of neurodegeneration.

Other studies have also examined the immune response associated with these pathogens. For example, Laugisch et al.^17^ observed the presence of antibodies against periodontal bacteria in the cerebrospinal fluid of patients with dementia, suggesting a possible intrathecal immune response to these microorganisms.

Furthermore, studies on the oral microbiome have demonstrated that changes in oral bacterial composition may be associated with AD. For example, analyses of the oral microbiome and serological markers have shown differences in the presence of periodontal bacteria between individuals with AD and healthy individuals.^20^

Experimental studies also provide important insights into the possible mechanisms underlying this relationship. Haditsch et al.^4^ demonstrated that *P. gingivalis* was capable of infecting human neurons in culture and inducing changes similar to those observed in AD, including tau protein degradation and synaptic loss. Furthermore, animal models have suggested that periodontal inflammation could promote increased Aβ deposits in the brain, which is a key feature of the disease.^3^

Similarly, Zhang et al.^11^ demonstrated that small RNA molecules produced by *P. gingivalis* can stimulate the production of Aβ in immune system cells, suggesting a possible molecular mechanism linking bacterial infection to AD pathology.

A study conducted in a murine model of AD^3^ demonstrated that experimental periodontitis can alter the inflammatory response in the brain. In this study, the induction of periodontitis led to changes in microglial cell activation and an increase in levels of insoluble Aβ_42_ in the brains of mice with AD-like pathology, suggesting that periodontal inflammation may influence neuroinflammatory processes and contribute to disease progression.

Several reviews have previously examined the association between periodontal disease and AD, including the putative role of *P. gingivalis*. Overall, these studies report convergent evidence suggesting a link between chronic periodontal infection, systemic inflammatory responses, and neurodegenerative processes. The originality of the present scoping review resides in both its methodological framework and its thematic focus. By adhering to the JBI and PRISMA-ScR recommendations, this review systematically maps the available evidence across epidemiological and clinical studies, as well as postmortem investigations and experimental models (in vitro and in vivo), with a specific emphasis on *P. gingivalis* rather than periodontal disease as a whole. Moreover, this study integrates more recent findings and provides an updated synthesis of the proposed mechanistic pathways, while explicitly identifying current knowledge gaps and methodological limitations, particularly regarding causal inference.

Despite these results, several limitations must be taken into account. Firstly, most of the available studies are observational or experimental and do not allow a direct causal link to be established between periodontal disease and AD.

Furthermore, these two conditions share several common risk factors, such as advanced age, diabetes smoking, systemic inflammation, comorbidities, or lifestyle factors, which may complicate the interpretation of the observed associations. Finally, findings regarding the direct presence of bacteria in the brain continue to vary across studies.

Although the present scoping review included a limited number of in vivo studies, this reflects the current state of research in this field, where much of the available evidence derives from in vitro experiments, postmortem analyses, and epidemiological or observational studies. This imbalance should be acknowledged when interpreting the findings. While experimental and observational data consistently suggest an association between *P. gingivalis* infection and mechanisms implicated in AD, such as neuroinflammation, Aβ accumulation, and tau pathology, these study designs do not allow firm conclusions regarding causality. In particular, as mentioned above, most human studies are associative in nature and may be influenced by shared risk factors. Therefore, the findings should be interpreted as supportive of a potential contributory role of *P. gingivalis* in AD pathophysiology rather than definitive evidence of a direct causal relationship.

Thus, although current evidence suggests that chronic infection with *P. gingivalis* may contribute to mechanisms involved in AD, further well-designed longitudinal studies and in vivo experimental models are required to clarify the temporal sequence and underlying mechanistic pathways, as well as to help define the potential impact of oral health on the risk of neurodegenerative diseases.

## Conclusion

The findings of this review suggest that there may be a link between infection with *P. gingivalis* and the development of AD. Several studies have shown that exposure to the bacteria responsible for periodontal disease, as well as the presence of antibodies against these bacteria, may be associated with a higher risk of developing cognitive impairment.

Some research has also identified the presence of bacterial components or virulence factors of *P. gingivalis* in the brains of patients with AD. Furthermore, experimental studies suggest that this bacterium may be involved in certain mechanisms observed in the disease, such as brain inflammation, alterations in tau protein, or increased production of Aβ.

However, the current findings do not yet allow us to conclude that there is a direct cause-and-effect relationship between periodontal disease and AD. In fact, AD is a complex condition that depends on many factors, including genetic, environmental and lifestyle-related factors.

Therefore, further research is needed to better understand the exact role of *P. gingivalis* in this disease. However, these findings highlight the importance of oral health and suggest that the prevention and treatment of periodontal disease may also have an impact on general health.
